# Effects of drought stress on growth, physiology and bioactive compound accumulation in licorice (*Glycyrrhiza* spp.): a meta-analysis-based integrated evaluation

**DOI:** 10.3389/fpls.2026.1931557

**Published:** 2026-08-28

**Authors:** Guoyin Li, Ruilan Yuan, Xiaoyan Li, Bi Zhang

**Affiliations:** 1 Fuxi Laboratory, College of Life Science and Agronomy, Zhoukou Normal University, Zhoukou, China; 2 Department of Hematology, Shanxi Provincial People’s Hospital, Taiyuan, China; 3 Department of Clinical Laboratory, Heping Branch, Shanxi Provincial People’s Hospital, Taiyuan, China

**Keywords:** antioxidant defense, bioactive compounds, drought stress, licorice, photosynthesis

## Abstract

**Introduction:**

Drought stress is a major environmental factor affecting the growth, physiological processes, and secondary metabolism of licorice (*Glycyrrhiza spp*.). However, the integrated responses of licorice to drought stress remain unclear.

**Methods:**

This meta-analysis synthesized evidence from multiple studies to evaluate the effects of drought stress on growth, physiology, and bioactive compounds in licorice. Random forest analysis and partial least squares path modeling (PLS-PM) were further applied to identify influential predictors and potential pathway relationships.

**Results:**

Drought significantly inhibited photosynthesis and growth. Net photosynthetic rate, stomatal conductance, and transpiration rate decreased by 38.31%, 27.74%, and 36.12%, respectively, while chlorophyll a, chlorophyll b, and SPAD values decreased by 20.83%, 27.52%, and 20.07%, respectively. Seed germination and shoot fresh weight were also reduced. Osmotic adjustment and antioxidant defenses were activated, with proline, soluble protein, and superoxide dismutase activity increasing by 79.82%, 41.30%, and 12.10%, respectively. However, malondialdehyde content increased by 52.90%, indicating insufficient protection against membrane lipid peroxidation. Bioactive compounds showed component-specific responses: total flavonoids increased by 14.97%, glycyrrhizic acid showed no significant overall change, and liquiritin declined in some subgroups. Drought intensity and soil water content were important predictors across physiological response categories, whereas treatment duration was particularly important for photosynthetic responses and bioactive compound accumulation.

**Discussion:**

Overall, licorice responses to drought involved photosynthetic inhibition, osmotic adjustment, antioxidant defense, and secondary metabolic reprogramming. Increases in individual phenolic compounds should not be interpreted as an overall improvement in medicinal quality, particularly when accompanied by photosynthetic inhibition, oxidative damage, and biomass loss.

## 1 Introduction

Licorice (*Glycyrrhiza* spp.) is a perennial medicinal plant belonging to the family Fabaceae and represents a major traditional Chinese medicinal resource. The principal medicinal species include *Glycyrrhiza uralensis, G. glabra, and G. inflata.* Their roots and rhizomes are rich in *glycyrrhizic* acid, liquiritin, flavonoids, polyphenols and triterpenoid saponins, supporting their broad application in pharmaceuticals, food and functional product development, and ecological restoration. In recent years, with the decline of wild resources and the expansion of cultivated production, increasing attention has been paid to the yield formation and quality stability of licorice, particularly in arid and semi-arid regions where water deficit has become a critical environmental constraint on seed germination, seedling establishment, root development and bioactive compound accumulation (Yao et al., 2022; Wang et al., 2023; Fozi et al., 2024). Previous studies have indicated that water deficit may promote the accumulation of certain bioactive compounds under some conditions, whereas prolonged or severe drought generally suppresses growth and exacerbates oxidative damage, suggesting that licorice responses to drought depend on stress intensity, treatment duration and the specific compound examined (Khaitov et al., 2021; Yue et al., 2022; Fozi et al., 2024).

Drought stress primarily affects leaf water status and gas-exchange processes by reducing soil water availability. Stomatal closure reduces transpirational water loss but simultaneously restricts CO_2_ entry into leaves, leading to a decrease in net photosynthetic rate and further affecting carbon assimilation and biomass accumulation. As stress intensifies, drought can also damage chloroplast structure and reduce photosynthetic pigment content and photosystem activity, causing photosynthetic limitation to shift from predominantly stomatal limitation to combined stomatal and non-stomatal limitations (Qiao et al., 2024). For licorice, different drought-imposition methods may induce distinct physiological responses. Soil-water-control treatments more closely mimic the gradual reduction in soil water availability under field conditions, whereas PEG treatments primarily simulate osmotic stress by reducing the water potential of the growth medium. Consequently, intercellular CO_2_ concentration, stomatal conductance and photosynthetic rate may respond differently between these two approaches. Therefore, it is necessary to distinguish the effects of drought-imposition method, drought intensity and experimental environment on the photosynthetic responses of licorice across a broader data range (Yao et al., 2022; Fozi et al., 2024).

Beyond the photosynthetic system, osmotic adjustment and antioxidant defense also represent important mechanisms by which licorice adapts to water deficit. Under drought conditions, plants often maintain cellular osmotic potential by accumulating proline, soluble sugars and soluble proteins, and scavenge excess reactive oxygen species through antioxidant enzymes such as SOD, POD and CAT. However, such protective responses may not completely prevent oxidative damage. When the rate of ROS production exceeds the scavenging capacity, membrane lipid peroxidation can still occur, manifesting as elevated MDA content (Hosseinifard et al., 2022; Urmi et al., 2023; Avendaño et al., 2025). Recent studies have also shown that proline functions not only as an osmotic regulator but also participates in redox homeostasis and stress-signal regulation. Nonetheless, the relationship between proline accumulation and drought tolerance is not simply linear, and comprehensive assessment still requires the integration of membrane-damage indicators with the coordinated responses of antioxidant enzymes (Ibrahim et al., 2022; Avendaño et al., 2025).

Drought can also reshape the secondary metabolic processes of medicinal plants. Metabolites such as flavonoids, polyphenols, terpenoids and saponins are often involved in antioxidant defense, photoprotection and defense-signal regulation, and their accumulation is generally influenced by stress intensity, treatment duration, organ type and species-specific genetic background (Yadav et al., 2021; Punetha et al., 2022; Bistgani et al., 2024). In licorice, PEG-induced drought can promote *glycyrrhizic* acid and flavonoid accumulation through JA-related signaling, and beneficial rhizosphere microorganisms may also alleviate drought-induced *glycyrrhizic* acid loss by modulating the JA pathway (Yao et al., 2022; Yue et al., 2022; Wang et al., 2023). However, findings from different studies on the responses of *glycyrrhizic* acid, liquiritin, total flavonoids and total phenols are not entirely consistent. Depending on the experimental conditions, the same compound may increase, decrease or remain unchanged. On the one hand, this inconsistency may relate to differences in licorice species and sampled organs; on the other hand, variations in drought intensity, treatment duration, experimental environment and exogenous regulatory measures may also contribute. Thus, a single experiment is insufficient to fully reveal the comprehensive effects of drought on licorice yield and quality.

Meta-analysis has been increasingly applied in ecology and plant science to quantitatively synthesize findings from independent studies. In addition to estimating the overall direction and magnitude of treatment effects, meta-analysis can evaluate between-study heterogeneity, assess the robustness of pooled results and identify moderator variables responsible for differences among studies (Borenstein et al., 2009; Gurevitch et al., 2018). This approach is particularly valuable for medicinal plants because environmental stress may simultaneously affect biomass production, physiological acclimation and the accumulation of different bioactive compounds. When combined with machine-learning and path-modeling approaches, meta-analysis can further help rank influential predictors, explore complex relationships and generate hypotheses for subsequent controlled experiments and field validation.

Despite the increasing number of studies on drought responses in licorice, three major issues remain unresolved. First, the reported direction and magnitude of changes in *glycyrrhizic* acid, liquiritin, total flavonoids and total phenols vary among studies, making it difficult to determine whether drought generally improves or reduces medicinal quality. Second, PEG-induced osmotic stress, soil-water-control treatments and field drought are not physiologically equivalent, yet their results are sometimes interpreted together without sufficient consideration of their distinct effects on plant physiology and the rhizosphere. Third, existing studies differ markedly in licorice species, sampled organs, developmental stages, drought intensities, treatment durations, experimental environments and exogenous treatments. These differences reduce cross-study comparability and may lead to conflicting recommendations for irrigation management, drought-tolerant germplasm selection and quality-oriented cultivation. These inconsistencies highlight the need for a quantitative synthesis that distinguishes major experimental moderators and evaluates growth, physiological responses and medicinal quality within a unified framework.

To address these issues, the present study systematically synthesized recent evidence on the effects of drought stress on the growth, physiology and bioactive compounds of licorice. Meta-analysis was used to quantify the overall effects of drought on photosynthetic parameters, photosynthetic pigments, osmotic adjustment substances, antioxidant responses, growth traits and medicinal compounds, while subgroup analyses compared responses across licorice species, experimental types, drought-imposition methods, drought intensities and exogenous treatments. Random forest and partial least squares path modeling (PLS-PM) were further used to identify influential predictors and potential pathway relationships. By integrating these approaches, this study aimed to clarify general and condition-dependent relationships among photosynthetic inhibition, growth restriction, defense activation, oxidative damage and secondary metabolic responses, and to provide an evidence base for standardized experimental design, water management, quality-oriented cultivation and drought-tolerant germplasm screening.

## 2 Data and methods

### 2.1 Data sources and extraction

We systematically searched Web of Science, PubMed, CNKI and Wanfang databases for studies published from database inception to April 2026 on the effects of drought stress on licorice. The search strategy was constructed as follows: (“*Glycyrrhiza*” OR “licorice”) AND (“drought” OR “water deficit” OR “PEG”).

The following inclusion criteria were established: (1) the research subject was *Glycyrrhiza* species, including *G. uralensis, G. glabra* and *G. inflata*; (2) the experimental group received drought stress treatment (including natural drought, water control, PEG-6000-simulated drought, etc.), while the control group was maintained under normal water supply under the same cultivation conditions; (3) at least one of the following indicators was reported: photosynthetic parameters (net photosynthetic rate Pn, stomatal conductance Gs, transpiration rate Tr, intercellular CO_2_ concentration Ci), chlorophyll content (chlorophyll a, chlorophyll b, SPAD values), the activities of antioxidant enzymes, including superoxide dismutase (SOD), peroxidase (POD), and catalase (CAT), oxidative damage indicator (MDA), osmotic adjustment substances (proline, soluble protein, soluble sugar), growth indicators (plant height, root length, shoot fresh weight, root fresh weight, germination potential, germination percentage), or bioactive compound contents (total flavonoids, liquiritin, *glycyrrhizic* acid, total phenols); (4) data were presented as means, standard deviations and sample sizes; (5) reviews, conference abstracts, duplicate publications and non-Chinese/English articles were excluded.

**Figure 1:**
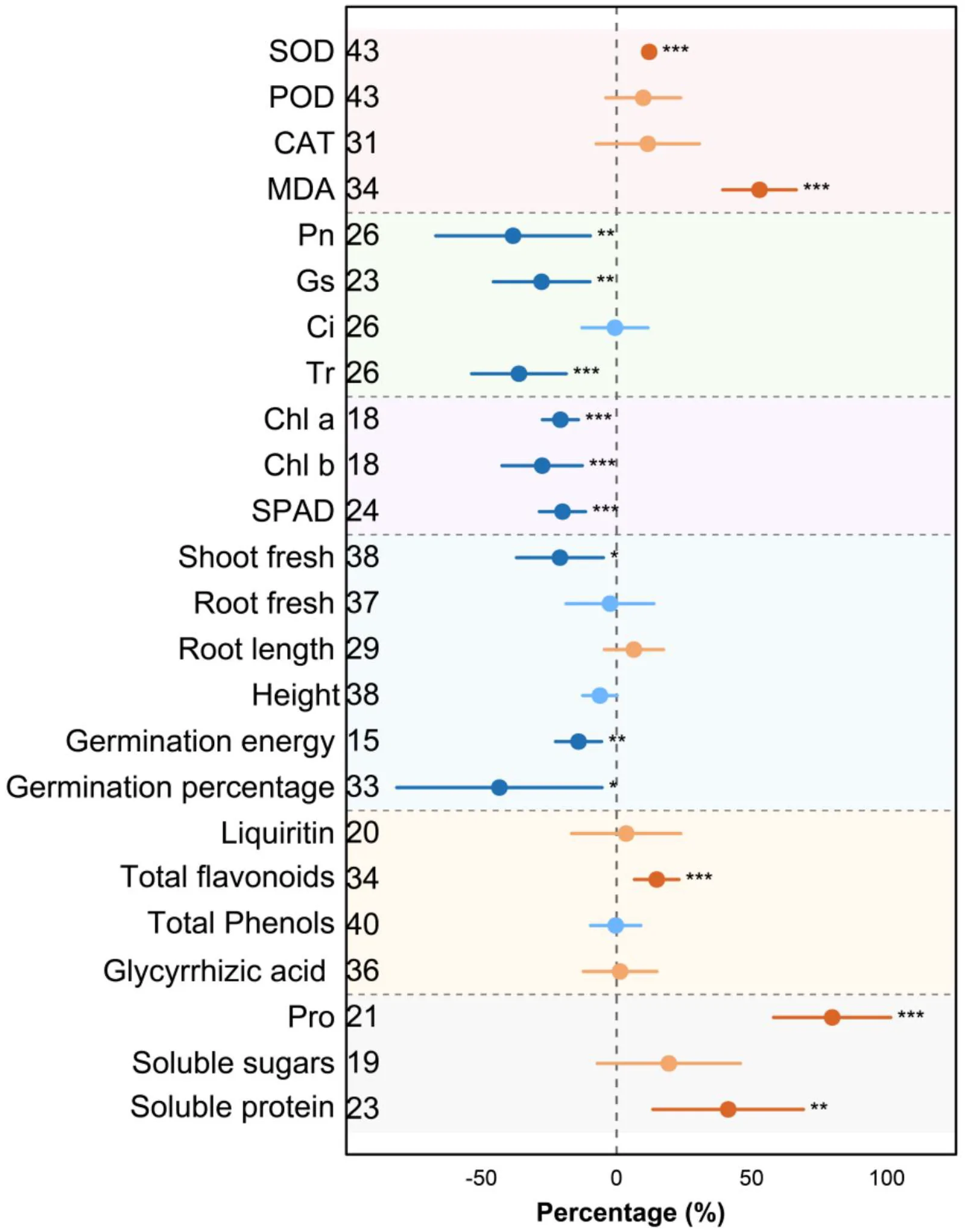
Overall effects of drought stress on growth, physiological traits, and bioactive compounds in licorice. Points represent the mean percentage change relative to the control, and horizontal bars indicate 95% confidence intervals. The vertical dashed line denotes no effect (0%). Numbers beside each variable indicate the number of effect sizes (*k*). Asterisks indicate statistical significance: *P < 0.05, **P < 0.01, and ***P < 0.001. SOD, superoxide dismutase; POD, peroxidase; CAT, catalase; MDA, malondialdehyde; Pn, net photosynthetic rate; Gs, stomatal conductance; Ci, intercellular CO_2_ concentration; Tr, transpiration rate; Chl a, chlorophyll a; Chl b, chlorophyll b; SPAD, soil plant analysis development value; Pro, proline.

The response ratio (RR) was used as the effect size metric, and paired data for the treatment and control groups were extracted from each included study. Data presented in tables were directly transcribed, while data presented in figures were digitally extracted using WebPlotDigitizer version 4.6. The literature screening process strictly followed PRISMA guidelines; the specific screening steps and inclusion/exclusion records are presented in **Supplementary Figure 1**. Following the systematic screening process (**Supplementary Figure 1**), a total of 31 peer-reviewed studies were included in the final meta-analysis, from which 696 paired comparisons (drought-treated vs. control) were extracted across all response variables. The number of observations for each specific indicator is presented in the corresponding forest plots (**Figure 1**).

### 2.2 Statistical analysis

Meta-analysis was performed using MetaWin 2.1 software (Sinauer Associates, Sunderland, MA, USA). Given the typically high heterogeneity in agroecological experiments, a random-effects model was adopted (Hedges et al., 1999; Borenstein et al., 2010). The effect size was expressed as the natural log-transformed RR, calculated as (Equation 1):

(1) $$\text{RR}=\text{In}(\frac{{\text{X}}_{\text{t}}}{{\text{X}}_{\text{c}}})=\text{ln}({\text{X}}_{\text{t}})-\text{ln}({\text{X}}_{\text{c}})$$

where Xt and Xc are the arithmetic means of the drought-treated and control groups, respectively. The variance (v) of RR was calculated according to equations (2–3) (Li et al., 2019a, Li et al., 2019b) (Equations 2, 3):

(2) $$v=\frac{{\text{SD}}_{\text{t}}^{2}}{{\text{n}}_{\text{t}}{\text{X}}_{\text{t}}^{2}}+\frac{{\text{SD}}_{\text{c}}^{2}}{{\text{n}}_{\text{c}}{\text{X}}_{\text{c}}^{2}}$$

(3) w=1/v

where SDt and SDc are the standard deviations of the treatment and control groups, respectively; nt and nc are the corresponding sample sizes; v is the variance of RR; and w is the weight assigned to RR. When studies reported standard errors (SE) rather than SD, conversion was performed according to equation (4) (Li et al., 2019a, Li et al., 2019b) (Equation 4):

(4) $$SD=\sqrt{\text{n}}\times SE$$

The weighted mean response ratio (RRw), its standard error (SE), and the 95% confidence interval (CI) were calculated as (Equations 5–7):

(5) $${\text{RR}}_{w}=\frac{\sum_{i=1}^{k}w_{i}{\text{RR}}_{i}}{\sum_{i=1}^{k}w_{i}}$$

(6) $$\text{SE}({\text{RR}}_{w})=\sqrt{\frac{1}{\sum_{i=1}^{k}w_{i}}}$$

(7) $$95\%\text{CI}={\text{RR}}_{w}\pm 1.96\text{SE}({\text{RR}}_{w})$$

An effect was considered statistically significant when the 95% CI did not overlap with zero (Calderón et al., 2000). A negative RR indicated that drought stress significantly decreased the corresponding indicator, whereas a positive RR indicated a significant increase. For ease of interpretation, RR values were converted to percentage changes induced by drought stress (Equation 8):

(8) $$\text{Percentage change }(\%)=(e^{RRw}-1)\times 100\%$$

Shapiro–Wilk normality tests indicated that the effect sizes did not follow a normal distribution (**Supplementary Figure 2**); therefore, bootstrap resampling (n = 4999) was used to estimate 95% confidence intervals (Shapiro and Wilk, 1965). Between-group heterogeneity was tested using the QB statistic and I² (**Supplementary Table 1**) (Light and Pillemer, 1984). Publication bias and result robustness were assessed using Begg’s test, Egger’s test and Rosenthal’s fail-safe number (Rosenthal, 1979; Begg and Mazumdar, 1994; Egger et al., 1997). Funnel plots were used to visualize the relationship between effect sizes and precision (**Supplementary Figure 3**).

### 2.3 Driving factor analysis based on machine learning and path modeling

To identify the key influencing factors and their potential relationships underlying the various physiological responses of licorice to drought stress, we employed an analytical framework combining random forest (RF) and PLS-PM. Random forest is an ensemble learning method based on classification and regression trees, which builds multiple decision trees and aggregates their predictions; it can handle complex data structures, identify non-linear relationships between predictors and response variables, and evaluate the relative contributions of predictors to model prediction performance (Breiman, 2001; Liaw and Wiener, 2002). This method has been widely applied to identify complex relationships between environmental factors and biological responses (Cutler et al., 2007).

**Figure 2:**
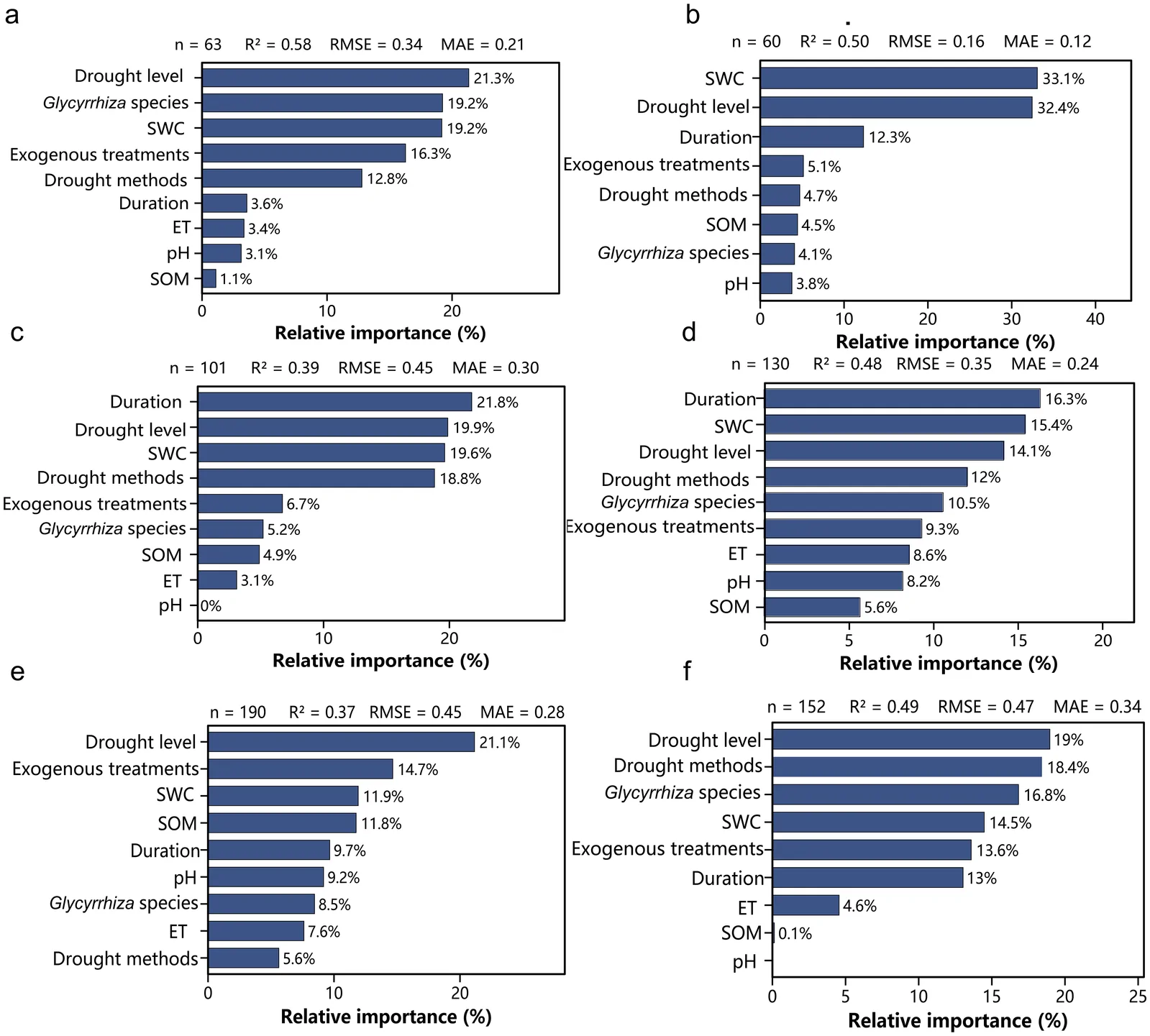
Relative importance of factors influencing licorice responses to drought stress based on random forest models. Panels show the relative importance of predictor variables for **(a)** osmotic adjustment substances, **(b)** photosynthetic pigments, **(c)** photosynthetic parameters, **(d)** bioactive compounds, **(e)** growth traits, and **(f)** oxidative stress-related parameters. Variable importance was evaluated according to the increase in root mean square error (RMSE) after permutation, with higher values indicating greater contributions to model prediction. Model performance is reported as sample size (*n*), coefficient of determination (R²), RMSE, and mean absolute error (MAE). SWC, soil water content; SOM, soil organic matter; ET, experimental type.

We constructed random forest models for six categories of response variables—photosynthetic parameters, photosynthetic pigments, osmotic adjustment substances, oxidative stress parameters, growth indicators and bioactive compounds—using licorice species, drought intensity, drought method, treatment duration, soil water content, soil organic matter and exogenous treatment as predictors. Ten-fold cross-validation was used to evaluate model prediction performance, and predictor importance was assessed by the increase in root mean square error (RMSE) of out-of-bag samples. Specifically, the RMSE of the original model on out-of-bag samples was first calculated; each predictor was then randomly permuted one at a time and the RMSE was recalculated. The larger the increase in RMSE after permutation, the greater the contribution of that predictor to model performance. Relative importance was then calculated and ranked for each variable to identify the key factors influencing different response indicators (**Figures 2A–F**).

**Figure 3:**
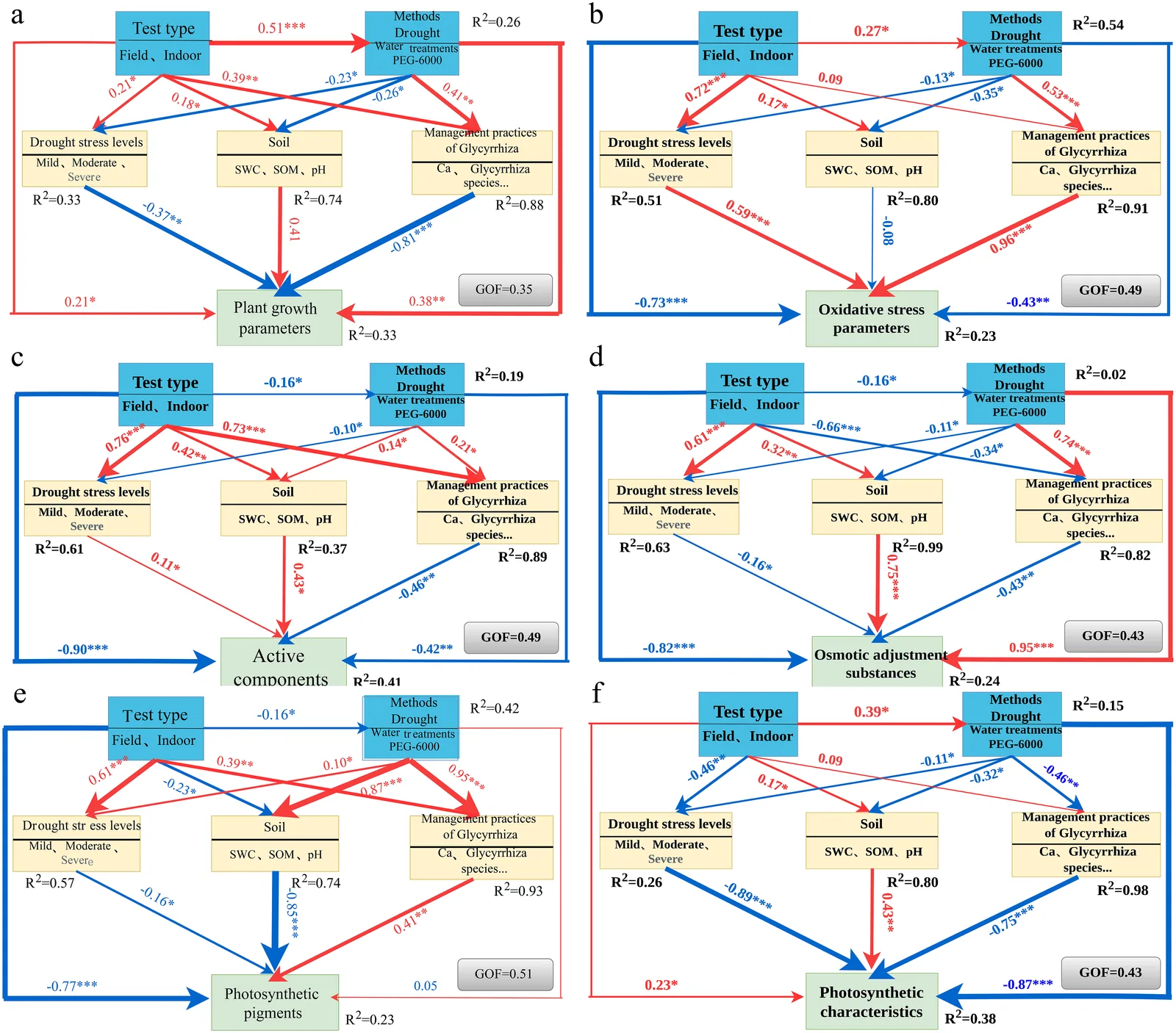
PLS-PM of the factors regulating licorice responses to drought stress. The models show the direct effects of experimental type, drought intensity, soil properties, drought-imposition method, and management-related factors on **(a)** plant growth parameters, **(b)** oxidative stress parameters, **(c)** bioactive compounds, **(d)** osmotic adjustment substances, **(e)** photosynthetic pigments, and **(f)** photosynthetic characteristics. Red and blue arrows indicate positive and negative effects, respectively, and arrow width is proportional to the absolute value of the path coefficient. Numbers beside the arrows represent standardized path coefficients. Asterisks indicate statistical significance: *P < 0.05, **P < 0.01, and ***P < 0.001. R² values indicate the variance explained for each endogenous variable, and GOF represents the overall goodness of fit. SWC, soil water content; SOM, soil organic matter; Ca, calcium treatment.

To further analyze the direct and indirect pathway relationships between influencing factors and licorice drought responses, we constructed PLS-PM. PLS-PM is a variance-based multivariate method that simultaneously estimates relationships between latent and observed variables, making it suitable for analyzing complex path relationships among multiple variables (Tenenhaus et al., 2005; Sanchez, 2013). In the model, predictors were grouped into latent variables representing drought intensity, soil factors, experimental type, drought method and management practices, and we analyzed their path coefficients and directions of effects on photosynthetic characteristics, photosynthetic pigments, osmotic adjustment substances, oxidative stress parameters, growth parameters and bioactive compounds. Overall model performance was described using the goodness-of-fit (GOF) index, combined with R² values of endogenous latent variables, path coefficients and their significance. Since GOF alone cannot validate PLS-PM models, we used it only as a descriptive indicator of overall model explanatory power (Henseler and Sarstedt, 2013). PLS-PM results are presented in **Figures 3A–F**.

All statistical analyses and graphical visualizations were performed using the following software and packages. Random forest modeling was implemented with the ‘randomForest’ package (version 4.7-1.1) in R version 4.2.3, with variable importance evaluated by permutation-based increases in root mean square error. Partial least squares path modeling was performed using the ‘plspm’ package (version 0.5.1) in R. All graphs were generated using the ‘ggplot2’ package (version 3.4.2) in R, with additional modifications in Adobe Illustrator 2023. Forest plots and subgroup figures were constructed using custom R scripts, and funnel plots were generated with the ‘meta’ package (version 6.2.1).

## 3 Results

### 3.1 Overall response of each indicator to drought stress

Drought stress significantly affected multiple physiological processes in licorice (**Figure 1**). For photosynthetic physiology, Pn, Gs and Tr decreased by 38.31%, 27.74% and 36.12%, respectively (*P* < 0.01*, P* < 0.01*, P* < 0.001); chlorophyll a, chlorophyll b and SPAD values decreased by 20.83%, 27.52% and 20.07%, respectively (all *P* < 0.001); while Ci showed no significant change. For antioxidant defense, SOD activity significantly increased by 12.10% (*P < 0.001*), and MDA content significantly increased by 52.90% (*P* < 0.001), indicating that drought induced oxidative stress and membrane lipid peroxidation damage, whereas POD and CAT activities showed increasing trends but did not reach statistical significance.

For osmotic regulation, proline and soluble protein contents significantly increased by 79.82% and 41.30%, respectively *(P* < 0.001*, P* < 0.01), while soluble sugar content did not change significantly. For growth indicators, shoot fresh weight significantly decreased by 20.90% (*P* < 0.05), and germination potential and germination percentage significantly decreased by 14.12% and 43.41%, respectively (*P* < 0.01*, P* < 0.05); root fresh weight, root length and plant height showed decreasing trends but did not reach statistical significance. For bioactive compounds, total flavonoid content significantly increased by 14.97% *(P* < 0.001), while liquiritin, total phenols and *glycyrrhizic* acid showed no significant changes.

### 3.2 Subgroup responses of photosynthetic traits and germination percentage to drought stress

**Figure 4:**
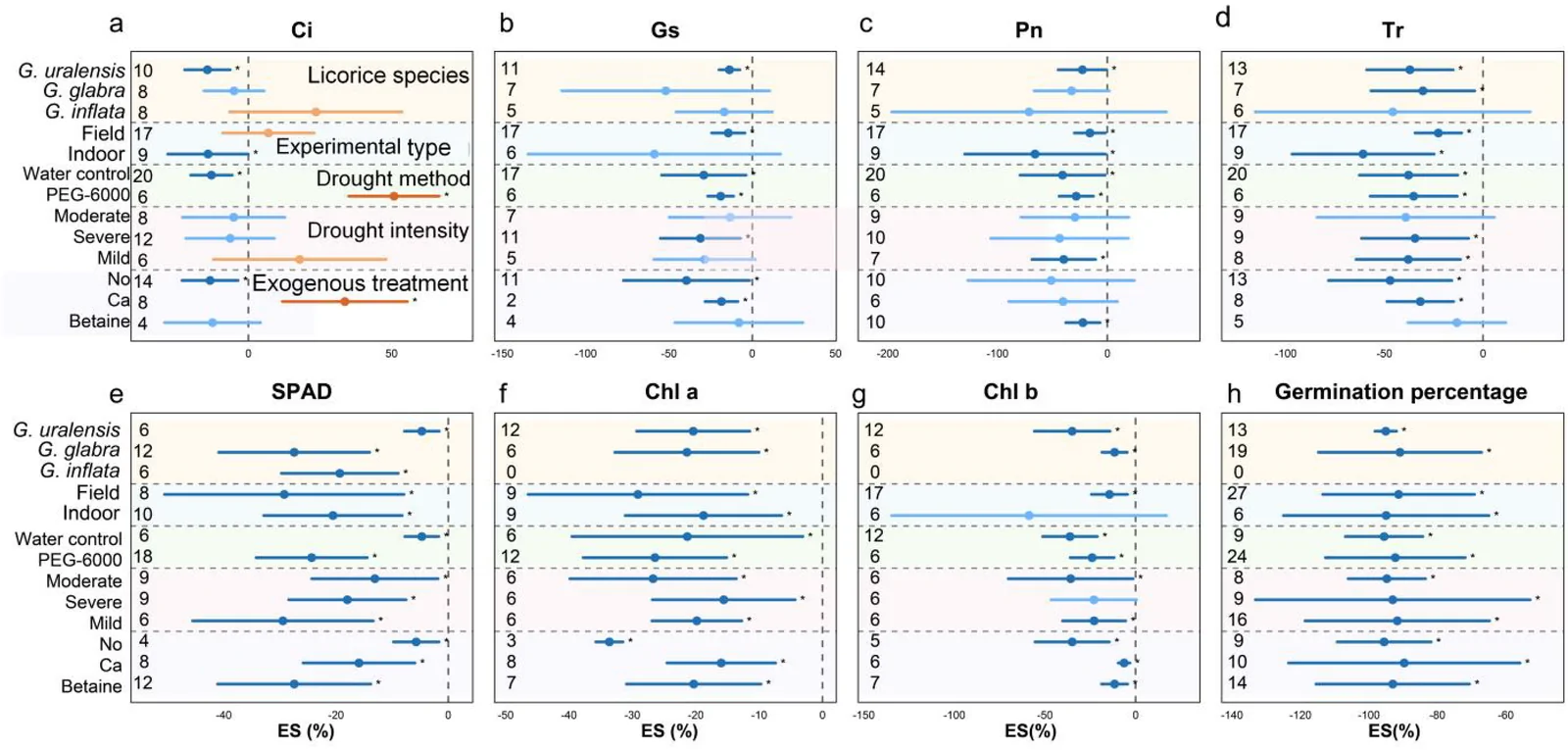
Subgroup meta-analysis of the effects of drought stress on photosynthetic traits and germination percentage in licorice. Forest plots show the pooled percentage changes in **(a)** intercellular CO_2_ concentration (Ci), **(b)** stomatal conductance (Gs), **(c)** net photosynthetic rate (Pn), **(d)** transpiration rate (Tr), **(e)** SPAD value, **(f)** chlorophyll a (Chl a), **(g)** chlorophyll b (Chl b), and **(h)** germination percentage. From top to bottom, subgroup levels are arranged according to licorice species, experimental types, drought-imposition method, drought intensity, and exogenous treatment. Horizontal dashed separators and alternating background shading distinguish the five moderator categories. Points represent weighted mean effect sizes expressed as percentage changes relative to the control, and horizontal lines represent 95% bootstrap confidence intervals. The vertical dashed line indicates no effect (ES = 0%). Numbers beside each subgroup indicate the number of effect sizes (*k*). Asterisks indicate significant effects: *P < 0.05,**P < 0.01, and ***P < 0.001. ES, effect size; PEG-6000, polyethylene glycol 6000; “No,” no exogenous treatment; Ca, calcium treatment; Betaine, glycine betaine treatment.

Subgroup heterogeneity tests (**Supplementary Table 1**) indicated that the responses of photosynthetic indicators differed significantly across subgroups (**Figures 4A–H**). The direction of change in Ci was significantly modulated by licorice species, drought method and exogenous treatment. In *G. uralensis*, Ci significantly decreased by 14.32% (*P < 0.05*), whereas under PEG-6000 and Ca treatments, Ci significantly increased by 50.88% and 33.71%, respectively (*P* < 0.05). The magnitude of reduction in Gs was significantly influenced by licorice species, experimental type and exogenous treatment: Gs decreased by 13.71% in *G. uralensis* (*P* < 0.05), and by 14.42%, 31.11% and 39.52% under field conditions, severe drought and no exogenous treatment, respectively (*P* < 0.05).

The decreases in Pn and Tr were mainly modulated by experimental type and exogenous treatment. The reduction in Pn was 65.89% under indoor conditions, significantly higher than the 15.90% observed under field conditions; Pn decreased by 40.80% under water control, compared with 22.26% under glycine betaine treatment (*P* < 0.05). Tr decreased by 22.64% and 60.77% under field and indoor conditions, respectively (*P* < 0.05), with reductions under water control, severe drought and Ca treatment ranging from 31.76% to 47.06% (*P* < 0.05).

The reduction in SPAD values was significantly modulated by licorice species, drought method, drought intensity and exogenous treatment. SPAD decreased by 27.46% and 19.33% in *G. glabra* and *G. inflata*, respectively, both greater than the 4.72% reduction in *G. uralensis*; the decrease under field conditions was 29.23%, higher than the 20.55% under indoor conditions (*P* < 0.05). Although chlorophyll a showed significant decreases within most subgroups, between-group heterogeneity was not significant (**Supplementary Table 1**), so subgroup comparisons were not performed.

Overall, drought stress primarily inhibited stomatal conductance, net photosynthetic rate, transpiration rate and photosynthetic pigment accumulation. The complex patterns of Ci change suggest that photosynthetic limitation in licorice under different conditions may involve both stomatal and non-stomatal factors.

### 3.3 Subgroup responses of growth indicators and osmotic adjustment substances to drought stress

**Figure 5:**
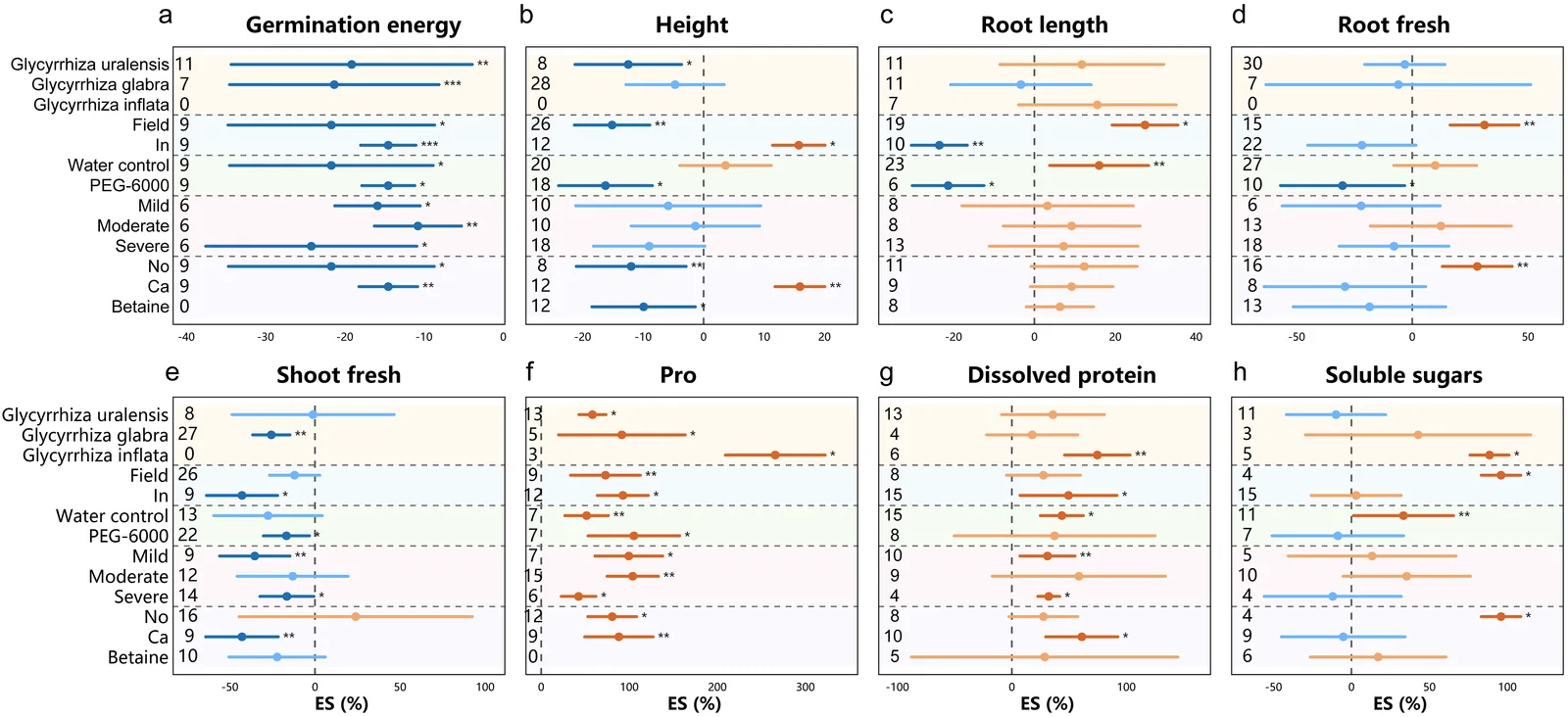
Subgroup analysis of the effects of drought stress on growth traits and osmotic adjustment substances in licorice. Forest plots show the percentage changes in **(a)** germination potential, **(b)** plant height, **(c)** root length, **(d)** root fresh weight, **(e)** shoot fresh weight, **(f)** proline content, **(g)** soluble protein content, and **(h)** soluble sugar content across licorice species, experimental conditions, drought-imposition methods, drought intensities, and exogenous treatments. Points represent mean effect sizes, and horizontal lines indicate 95% confidence intervals. The vertical dashed line denotes no effect (0%). Numbers beside each subgroup indicate the number of effect sizes (*k*). Asterisks indicate significant effects:*P < 0.05,**P < 0.01, and ***P < 0.001. “No” indicates no exogenous treatment; Ca, calcium treatment; Betaine, betaine treatment.

Subgroup heterogeneity tests (**Supplementary Table 1**) showed that the responses of growth and osmotic adjustment indicators to drought exhibited selective differences across subgroups (**Figures 5A–H**). The reduction in germination potential was significantly affected by experimental type, drought method, drought intensity and exogenous treatment. Germination potential decreased by 21.69% and 14.50% under field and indoor conditions, respectively (*P* < 0.05, *P* < 0.001); under mild, moderate and severe drought, it decreased by 15.87%, 10.77% and 24.22%, respectively (*P* < 0.05, *P* < 0.01, *P* < 0.05), with the largest reduction under severe drought.

Plant height was significantly modulated by experimental type, drought method and exogenous treatment. Under field conditions and PEG-6000 treatment, plant height decreased by 15.12% and 16.21%, respectively (*P* < 0.01, *P* < 0.05), whereas under indoor conditions and Ca treatment, it significantly increased by 15.74% and 15.93%, respectively (*P* < 0.05, *P* < 0.01). Root length significantly increased under field conditions and water control, but significantly decreased under indoor conditions and PEG-6000 treatment; root fresh weight significantly increased only under field conditions and without exogenous treatment (*P* < 0.01). The reduction in shoot fresh weight was significantly influenced by licorice species, experimental type and exogenous treatment: it decreased by 25.64% in *G. glabra* (*P* < 0.01), and by 42.87% under both indoor conditions and Ca treatment (*P* < 0.05, *P* < 0.01).

For osmotic adjustment substances, proline accumulation was significantly affected by licorice species, increasing by 58.25%, 91.61% and 265.76% in *G. uralensis*, *G. glabra* and *G. inflata*, respectively (*P* < 0.05). Soluble sugar content was significantly modulated by licorice species and experimental type, increasing by 88.59% and 95.93% in *G. inflata* and under field conditions, respectively (*P* < 0.05, *P* < 0.001). Soluble protein showed no significant between-group heterogeneity across any subgroup (**Supplementary Table 1**), so subgroup comparisons were not performed.

Overall, drought stress significantly reduced germination potential, plant height and shoot fresh weight in licorice, while the effects on root growth varied with experimental conditions and drought method. The enhanced accumulation of proline and soluble sugars suggests that licorice can alleviate physiological pressure from water deficit through osmotic adjustment.

### 3.4 Subgroup responses of bioactive compounds and oxidative stress parameters to drought stress

**Figure 6:**
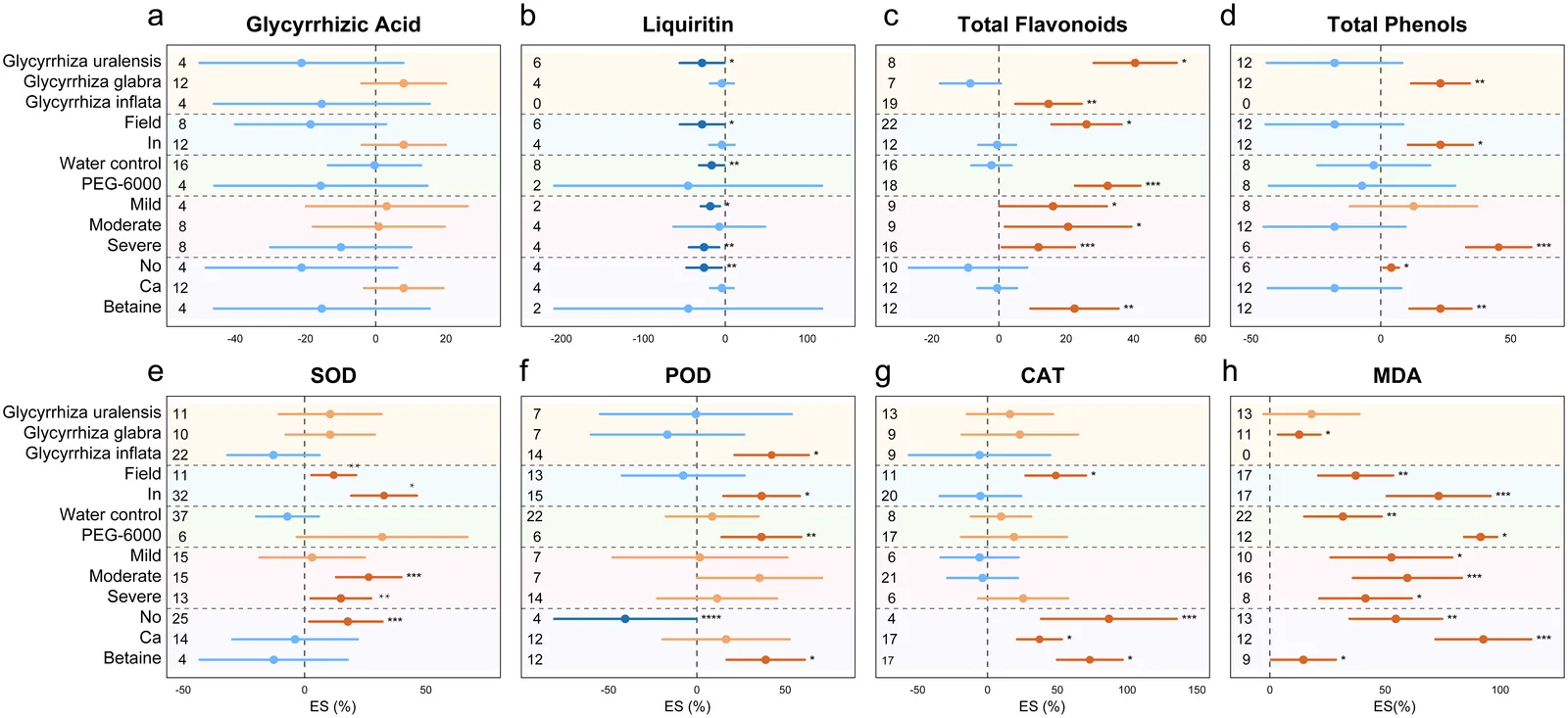
Subgroup analysis of the effects of drought stress on bioactive compounds and oxidative stress-related parameters in licorice. Forest plots show the percentage changes in **(a)** *glycyrrhizic* acid, **(b)** liquiritin, **(c)** total flavonoids, **(d)** total phenols, **(e)** superoxide dismutase activity (SOD), **(f)** peroxidase activity (POD), **(g)** catalase activity (CAT), and **(h)** malondialdehyde content (MDA) across licorice species, experimental conditions, drought-imposition methods, drought intensities, and exogenous treatments. Points represent mean effect sizes, and horizontal lines indicate 95% confidence intervals. The vertical dashed line denotes no effect (0%). Numbers beside each subgroup indicate the number of effect sizes (*k*). Asterisks indicate statistical significance: *P < 0.05,**P < 0.01, and ***P < 0.001. “No” indicates no exogenous treatment; Ca, calcium treatment; Betaine, betaine treatment.

Subgroup heterogeneity tests (**Supplementary Table 1**) indicated that the responses of bioactive compounds and oxidative stress parameters were modulated by multiple subgroup factors (**Figures 6A–H**). Among bioactive compounds, *glycyrrhizic* acid showed no significant change across all subgroups. Liquiritin mainly showed a decreasing trend, with a significant reduction of 28.29% under field conditions (P < 0.05). Total flavonoid content generally increased and was significantly modulated by licorice species, drought method and exogenous treatment: total flavonoids increased by 40.51% and 14.75% in *G. uralensis* and *G. inflata*, respectively (*P* < 0.05, *P* < 0.01), and by 32.35% and 22.47% under PEG-6000 and glycine betaine treatments, respectively (*P* < 0.001, *P* < 0.01). Total phenols increased by 22.99% in *G. glabra*, under indoor conditions and with glycine betaine treatment (*P* < 0.01), but decreased by 7.22% under PEG-6000 treatment (*P* < 0.001).

Overall, drought stress promoted total flavonoid accumulation and significantly elevated MDA content, indicating that licorice undergoes both secondary metabolic regulation and membrane lipid peroxidation damage under drought conditions. The direction of changes in different antioxidant enzyme activities was not consistent, suggesting that their responses are jointly influenced by licorice species, drought conditions and exogenous treatments.

### 3.5 Key driving factors and pathway analysis

Machine learning results showed that different categories of indicators were dominated by different factors in their responses to drought stress (**Figures 2A–F**). Overall, drought intensity and soil water content were the core factors affecting all categories. Photosynthetic pigments were mainly influenced by soil water content (33.1%) and drought intensity (32.4%); growth indicators were most strongly affected by drought intensity (21.1%); treatment duration contributed most to photosynthetic indicators (21.8%), followed by drought intensity, soil water content and drought method. Osmotic adjustment substances were jointly influenced by drought intensity (21.3%), licorice species (19.2%) and soil water content (19.2%); bioactive compounds were regulated by multiple factors with contributions ranging from 14% to 16%; oxidative stress parameters were mainly influenced by drought intensity (19.0%), drought method (18.4%) and licorice species (16.8%). Thus, drought intensity and soil water content were the most universally important factors, duration primarily influenced photosynthesis and bioactive compound accumulation, and licorice species played a prominent role in osmotic adjustment and oxidative stress responses.

PLS-PM path analysis further revealed the potential pathway relationships between various factors and licorice drought responses (**Figures 3A–F**). The GOF values of the six models ranged from 0.35 to 0.51, with the R² of each indicator ranging from 0.23 to 0.41. Plant growth parameters were significantly positively affected by soil factors (0.41, *P* < 0.05) and negatively affected by drought intensity (−0.37, *P* < 0.01) and management practices (−0.81, *P* < 0.001); oxidative stress parameters were positively affected by drought intensity (0.59, *P* < 0.001) and management practices (0.96, *P* < 0.001). Bioactive compounds were positively affected by soil factors (0.43, *P* < 0.05) and drought intensity (0.11, *P* < 0.05), while experimental type showed the strongest negative effect (−0.90, *P* < 0.001); osmotic adjustment substances were mainly positively affected by drought method (0.95, *P* < 0.001). Photosynthetic pigments and photosynthetic characteristics were both significantly affected by experimental type and soil factors, with photosynthetic characteristics additionally subject to significant negative effects from drought intensity (−0.89), drought method (−0.87) and management practices (−0.75) (all *P* < 0.001). Overall, drought intensity, soil conditions, drought method and management practices jointly influence the physiological responses of licorice, with drought primarily inhibiting growth and photosynthetic processes while promoting oxidative stress and partial secondary metabolic responses.

## 4 Discussion

### 4.1 Drought-induced stomatal and non-stomatal limitations constrain photosynthesis and alter growth allocation

Under drought stress, licorice first exhibits photosynthetic system suppression, which subsequently leads to growth reduction. In the present study, Pn, Gs and Tr decreased by 27.74%–38.31%, while chlorophyll a, chlorophyll b and SPAD values decreased concurrently, and germination potential, germination percentage and shoot fresh weight were significantly inhibited. This overall trend is broadly consistent with the established understanding that water deficit induces stomatal closure, restricts CO_2_ diffusion, and consequently reduces photosynthetic carbon assimilation and growth (Flexas and Medrano, 2002; Chaves et al., 2009; Lawlor and Tezara, 2009). In other words, drought does not affect individual photosynthetic indicators in isolation, but rather produces a continuous inhibitory cascade from gas exchange through pigment accumulation to biomass formation.

Nevertheless, subgroup analyses revealed a notable difference: Ci showed no overall significant change, but increased significantly by 50.88% under PEG-6000 treatment (*P* < 0.05), while tending to decrease under soil drought (**Figure 4A**). This divergence aids in diagnosing the type of photosynthetic limitation. When Ci decreases along with Gs, the photosynthetic limitation is more likely to be stomatal; when Ci increases while Pn decreases, it suggests that although CO_2_ enters the leaf, it is not effectively fixed, and non-stomatal limitation may dominate, such as chloroplast structural damage, reduced Rubisco carboxylation efficiency or impaired electron transport (Cornic, 2000; Flexas et al., 2004; Pinheiro and Chaves, 2011). Therefore, the difference in Ci response direction between PEG-6000-simulated osmotic stress and soil drought does not represent conflicting results, but rather indicates that the mechanisms of photosynthetic limitation differ across drought imposition methods.

Regarding photosynthetic pigments, chlorophyll a, chlorophyll b and SPAD values generally decreased, indicating that drought not only restricts stomatal opening but also damages the light-harvesting system (**Figures 4E–G**). Water deficit can induce ROS accumulation, which in turn damages chloroplast membrane structure and pigment-protein complexes, accelerating chlorophyll degradation (Farooq et al., 2009; Osakabe et al., 2014; Dong et al., 2024). Machine learning results support this interpretation: photosynthetic pigments were mainly driven by soil water content and drought intensity, with relative importance of 33.1% and 32.4%, respectively. PLS-PM further showed that drought intensity and drought method had significant negative effects on photosynthetic characteristics, while soil factors exerted positive buffering effects. This suggests that drought intensity likely indirectly limits Pn primarily by impairing photosynthetic pigment accumulation and gas exchange capacity, ultimately leading to reduced shoot growth.

This photosynthetic inhibition further alters growth allocation in licorice (Eziz et al., 2017). In the present study, shoot fresh weight significantly decreased by 20.9% (*P* < 0.05), while root length increased under field conditions and water control (**Figure 5C**). This indicates that licorice may prioritize root elongation under water deficit, allocating limited carbon resources to belowground parts to enhance water acquisition capacity. Similarly, Han et al. found significant interspecific differences in germination, physiological tolerance and bioactive compound responses among three licorice species under drought, but root adaptation and aboveground inhibition were common response directions (Han et al., 2022; Zhang et al., 2017, Zhang et al., 2018). Therefore, water management during the seedling stage of licorice should focus on sensitive indicators such as SPAD, Gs and Pn, and avoid continuous drought that causes photosynthetic system damage during germination and early seedling stages.

### 4.2 Osmotic adjustment and antioxidant defenses are insufficient to fully offset drought-induced oxidative membrane damage

Under drought stress, licorice is not merely damaged but concurrently activates osmotic adjustment and antioxidant defense. In the present study, proline and soluble protein both increased significantly; in subgroup analyses, proline in *G. inflata* increased by as much as 265.76%, and proline accumulation was also evident under PEG-6000 treatment and moderate drought (**Figure 5F**). This indicates that proline is a relatively stable osmotic adjustment indicator in licorice’s response to drought. Proline is known not only to maintain cellular osmotic potential but also to stabilize proteins and membrane structures and participate in redox balance regulation (Verbruggen and Hermans, 2008; Szabados and Savouré, 2010; Hayat et al., 2012). The increase in soluble protein may be related to enhanced synthesis of stress-related proteins such as LEA proteins and heat shock proteins, which help maintain enzyme activity and cellular hydration status (Battaglia et al., 2008; Wang et al., 2004).

However, enhanced protective responses do not imply complete alleviation of oxidative damage. In the present study, MDA content significantly increased overall, and was broadly elevated under water control, PEG-6000 and across different drought intensities (**Figure 6H**). This indicates that although licorice initiated defense responses, membrane lipid peroxidation continued to accumulate (Li and Wang, 2002; Pan et al., 2006). The reason may lie in the functional division and asynchronous responses of the antioxidant enzyme system. SOD primarily converts O_2_^-^ to H_2_O_2_, while POD and CAT are responsible for further H_2_O_2_ scavenging; if SOD activity increases but POD and CAT do not respond synchronously, H_2_O_2_ may continue to accumulate and generate more reactive ·OH through the Fenton reaction, ultimately exacerbating membrane lipid peroxidation (Apel and Hirt, 2004; Gill and Tuteja, 2010; Hasanuzzaman et al., 2020).

Subgroup results also indicate that antioxidant responses are condition-dependent (**Figures 6E–H**). POD significantly increased in G. inflata, under indoor conditions and with glycine betaine treatment; CAT significantly increased without exogenous treatment and with Ca and glycine betaine treatments (Dong et al., 2024), suggesting that POD and CAT activation may require stronger stress signals, longer response times, or specific exogenous regulation conditions. Compared with studies showing that exogenous Ca, glycine betaine or silicon can reduce MDA, in the present study MDA remained elevated under some exogenous treatments, likely related to variations in drought intensity, treatment concentration, duration and licorice species across the included studies (Zhang et al., 2017, Zhang et al., 2018). Therefore, whether exogenous regulation truly alleviates drought damage cannot be judged solely by increased antioxidant enzyme activity; it must also be assessed alongside whether MDA declines (Li et al., 2003; Li et al., 2004; Zhang et al., 2020).

Machine learning and PLS-PM results further reveal the driving factors behind these processes (**Figures 2**, **3**). Osmotic adjustment substances were mainly influenced by drought intensity, licorice species and soil water content, while oxidative stress parameters were mainly driven by drought intensity, drought method and licorice species. PLS-PM showed that drought intensity had positive effects on oxidative stress parameters, indicating that stronger drought more readily triggers oxidative stress responses in licorice, but also concomitantly increases the risk of membrane system damage. This suggests that drought tolerance in licorice does not depend on the accumulation of any single protective compound, but rather on the balance among osmotic adjustment capacity, antioxidant enzyme synergies and membrane lipid peroxidation levels (Wahab et al., 2022; Renzetti et al., 2024). Therefore, drought-tolerant germplasm screening should simultaneously consider proline accumulation, MDA levels, and the coordinated responses of SOD, POD and CAT, prioritizing materials with strong osmotic adjustment, low oxidative damage and well-coordinated antioxidant systems.

### 4.3 Drought induces secondary metabolic reprogramming, but quality enhancement carries risks of yield and component stability

Drought stress induces secondary metabolic reprogramming in licorice, but the direction and magnitude of the responses vary among medicinal components (Jangpangi et al., 2025;Muthusamy and Lee, 2024; Zhang et al., 2022). In the present study, total flavonoid content increased significantly by 14.97% (*P* < 0.001), and increasing trends were observed in *Glycyrrhiza uralensis*, *G. inflata*, under field conditions, under PEG-6000 treatment, and across different drought intensities. Subgroup analysis further showed that, in some comparisons, the increases in total flavonoids and total phenols were greater under severe drought than under moderate drought. This suggests that stronger drought may more markedly activate the defensive metabolism of certain phenolic compounds; however, this response is compound- and condition-specific and does not indicate that severe drought universally improves the medicinal quality of licorice. Flavonoids possess reactive oxygen species-scavenging, photoprotective, and membrane-stabilizing functions, and their accumulation is widely regarded as a typical response to activation of the phenylpropanoid pathway under drought, high-light, and oxidative stress (Dixon and Paiva, 1995; Winkel-Shirley, 2002; Nakabayashi et al., 2014). Therefore, the simultaneous increases in total flavonoids and MDA observed in this study are not contradictory; rather, they indicate that licorice enhances defensive secondary metabolism while experiencing oxidative damage (Hosseini et al., 2018).

However, not all bioactive compounds increased with increasing drought severity. *Glycyrrhizic* acid remained largely stable in the overall analysis and across subgroups, whereas liquiritin showed a decreasing trend under several conditions. Therefore, the greater increases in total flavonoids and total phenols under severe drought cannot be extrapolated to *glycyrrhizic* acid or liquiritin, nor can an increase in a single class of phenolic compounds be used to infer an overall improvement in medicinal quality. These contrasting responses indicate that flavonoids, flavonoid glycosides, and triterpenoid saponins differ in their sensitivity and metabolic responses to drought signals (Nasrollahi et al., 2014). Flavonoids are directly involved in antioxidant defense and may respond rapidly to stress signals such as ROS, abscisic acid (ABA), and jasmonic acid (JA) (Jogawat, 2021). By contrast, *glycyrrhizic* acid, as a triterpenoid saponin, may depend more strongly on sustained photosynthetic carbon supply, root development, and organ-specific accumulation processes (Ramakrishna and Ravishankar, 2011; Isah, 2019; Yeshi et al., 2022). This may explain why total flavonoids generally increased, whereas *glycyrrhizic* acid remained largely stable.

The decline in liquiritin content may reflect a redistribution of metabolic flux under drought conditions. Phenylpropanoid precursors may be preferentially directed toward flavonol or chalcone branches with stronger antioxidant functions, whereas the synthesis of dihydroflavone glycosides may be constrained by glycosyltransferase activity, UDP-sugar donor availability, or insufficient carbon supply. However, this interpretation remains a mechanistic hypothesis based on the current findings and cannot be directly confirmed by the correlational analyses used in this study. Further validation through metabolomic, transcriptomic, and key enzyme activity analyses is therefore required. Previous studies have also shown that plants under stress often prioritize the accumulation of defensive secondary metabolites, although the responses of different metabolic branches are jointly influenced by species, organ type, and treatment duration (Treutter, 2006; Falcone Ferreyra et al., 2021; Sharma et al., 2019).

In contrast to the findings of Yao et al., who observed simultaneous increases in *glycyrrhizic* acid and total flavonoids under PEG-6000 treatment, *glycyrrhizic* acid did not change significantly in the present study. This discrepancy may be attributable to two factors. First, Yao et al. primarily used short-term PEG-6000 simulation, whereas the present study integrated data from field and indoor experiments, soil-water-control and PEG treatments, and treatments of different durations, resulting in greater environmental and methodological heterogeneity. Second, different metabolic pathways may have different response thresholds. Flavonoids, as rapidly inducible defensive metabolites, may respond more readily to drought signals, whereas *glycyrrhizic* acid accumulation may require a longer time scale or a more stable carbon supply to the roots (Han et al., 2022; Yao et al., 2022; Yue et al., 2022). Therefore, our results do not exclude the possibility that drought promotes *glycyrrhizic* acid accumulation under specific conditions, but rather indicate that such promotion is not consistent across studies and treatment conditions.

Machine-learning results showed that variation in bioactive compounds was closely associated with treatment duration, soil water content and drought intensity. PLS-PM results indicated positive associations of soil factors and drought intensity with bioactive compounds, whereas experimental types, drought-imposition method, and management practices may exert negative effects (**Figure 2**–6). Overall, our findings do not support the existence of a single optimal drought intensity applicable to all licorice germplasms and target compounds. Although the increases in total flavonoids and total phenols were greater under severe drought than under moderate drought in some subgroup estimates, severe drought did not simultaneously promote *glycyrrhizic* acid accumulation, and liquiritin declined under some conditions. In addition, stronger or prolonged drought may reduce photosynthetic carbon assimilation, inhibit biomass formation, and aggravate membrane lipid peroxidation. Therefore, increases in phenolic compounds should be regarded as defensive metabolic responses rather than being equated with a comprehensive improvement in medicinal quality.

In production practice, short-term or moderate water deficit should not be broadly recommended as a universal strategy for improving licorice quality. Water management should be optimized according to the target compound, licorice germplasm, developmental stage, and acceptable yield loss, with simultaneous assessment of root yield, *glycyrrhizic* acid, liquiritin, total flavonoids, total phenols, and MDA. Controlled water deficit may have practical value only when gains in bioactive compounds are sufficient to offset the risks of biomass loss and oxidative damage.

### 4.4 Limitations

This study integrated the growth, physiological and bioactive-compound responses of licorice to drought through meta-analysis and complemented the synthesis with random forest and PLS-PM. Several limitations should nevertheless be acknowledged. The included studies differed in licorice species, drought-imposition methods, stress intensities, treatment durations and experimental environments, while sample sizes for *G. inflata*, glycine betaine treatments and several bioactive-compound indicators were relatively small. Therefore, some subgroup estimates should be interpreted as indicative trends and validated using independent datasets (Borenstein et al., 2009; Gurevitch et al., 2018). However, this heterogeneity also provided informative between-study variation, enabling subgroup and machine-learning analyses to identify condition-dependent patterns and generate testable hypotheses for future studies.

PEG-6000 primarily simulates osmotic stress and cannot fully reproduce field drought, which also involves changes in soil structure, nutrient availability, rhizosphere conditions and microbial processes. Therefore, increased intercellular CO_2_ concentration accompanied by reduced net photosynthetic rate under PEG treatment can only suggest enhanced non-stomatal limitation (Verslues et al., 2006; Flexas and Medrano, 2002; Chaves et al., 2009; Pinheiro and Chaves, 2011). In addition, random forest and PLS-PM identify variable importance and potential pathway associations but cannot establish causality (Breiman, 2001; Sanchez, 2013; Pearl, 2010). Future studies should therefore apply standardized drought gradients, quantitatively report soil water status and treatment duration, and distinguish PEG-induced osmotic stress from soil drought. The validation of the identified associations with independent datasets, field trials and multi-omics approaches is also needed.

## 5 Conclusions

This study demonstrates that integrating meta-analysis with random forest and PLS-PM can effectively synthesize large, heterogeneous datasets and identify both general and condition-dependent patterns in licorice responses to drought. Overall, drought inhibited gas exchange, photosynthetic pigment accumulation, seed germination and aboveground growth. Although osmotic adjustment and antioxidant defenses were activated, the persistent increase in MDA indicated incomplete protection against oxidative membrane damage. Bioactive compounds showed component-specific responses: total flavonoids generally increased, and some subgroup estimates showed greater increases in total flavonoids and total phenols under severe than moderate drought, whereas *glycyrrhizic* acid remained largely unchanged and liquiritin declined under several conditions. Therefore, increases in individual phenolic compounds should not be interpreted as an overall improvement in medicinal quality when accompanied by photosynthetic inhibition, oxidative damage and biomass loss.

No single drought regime was consistently optimal across compounds or licorice germplasms. Future studies should standardize drought gradients, quantitatively report soil water status and treatment duration, distinguish PEG-induced osmotic stress from soil drought, and simultaneously assess root yield, physiological damage and multiple bioactive compounds across genotypes and developmental stages. Random-forest variable rankings and PLS-PM-derived associations should be validated using independent datasets, long-term field trials and multi-omics analyses. Water management should therefore be tailored to the target compound, germplasm and production objective.

## Data Availability

The raw data supporting the conclusions of this article will be made available by the authors, without undue reservation.
