# Supplementary material for: Effects of drought stress on growth, physiology and bioactive compound accumulation in licorice (Glycyrrhiza spp.): a meta-analysis-based integrated evaluation
Source: Front Plant Sci. 2026 Aug 28;17:1931557. doi: 10.3389/fpls.2026.1931557 (PMC13561827; doi:10.3389/fpls.2026.1931557)
Supplement: Supplementary file 1 [file SupplementaryFile1.docx]

Supplementary Material

**Figure. S1**: The PRISMA flow diagram. “n” is the number of studies.

**Figure. S2** Gaussian fitted histogram plotted using effect values (RR)

**Figure. S3.** Funnel plots for publication bias .

**Table S1. Subgroup heterogeneity analysis of drought effects on Glycyrrhiza indicators**

| **Variable** | **Subgroup** | **QB** | **P** | **I²** |
| --- | --- | --- | --- | --- |
| Ci | Licorice species | 9.0139 | 0.0110 | 77.81% |
|  | Experiment type | 2.8445 | 0.0917 | 64.84% |
|  | Drought method | 39.7110 | < 0.0001 | 97.48% |
|  | Drought intensity | 2.0949 | 0.3508 | 4.53% |
|  | ExtraTreatment type | 21.9947 | < 0.0001 | 90.91% |
| Gs | Licorice species | 31.8164 | < 0.0001 | 93.71% |
|  | Experiment type | 48.9244 | < 0.0001 | 97.96% |
|  | Drought method | 2.1491 | 0.1426 | 53.47% |
|  | Drought intensity | 5.2728 | 0.0716 | 62.07% |
|  | ExtraTreatment type | 15.9625 | 0.0011 | 81.21% |
| Pn | Licorice species | 28.3490 | < 0.0001 | 92.95% |
|  | Experiment type | 42.8628 | < 0.0001 | 97.67% |
|  | Drought method | 0.9801 | 0.3222 | 0.00% |
|  | Drought intensity | 1.1113 | 0.5737 | 0.00% |
|  | ExtraTreatment type | 8.6231 | 0.0134 | 76.81% |
| Tr | Licorice species | 1.7659 | 0.4136 | 0.00% |
|  | Experiment type | 27.6319 | < 0.0001 | 96.38% |
|  | Drought method | 0.0788 | 0.7789 | 0.00% |
|  | Drought intensity | 0.2406 | 0.8867 | 0.00% |
|  | ExtraTreatment type | 9.8622 | 0.0072 | 79.72% |
| SPAD | Licorice species | 12.1645 | 0.0023 | 83.56% |
|  | Experiment type | — | — | — |
|  | Drought method | 9.8938 | 0.0017 | 89.89% |
|  | Drought intensity | 13.4088 | 0.0012 | 85.08% |
|  | ExtraTreatment type | 9.8802 | 0.0072 | 79.76% |
| Chlorophyll a | Licorice species | 0.0314 | 0.8593 | 0.00% |
|  | Experiment type | — | — | — |
|  | Drought method | — | — | — |
|  | Drought intensity | 4.7652 | 0.0923 | 58.03% |
|  | ExtraTreatment type | 4.9897 | 0.0825 | 59.92% |
| Chlorophyll b | Licorice species | 10.6629 | 0.0011 | 90.62% |
|  | Experiment type | — | — | — |
|  | Drought method | — | — | — |
|  | Drought intensity | 2.2517 | 0.3244 | 11.18% |
|  | ExtraTreatment type | 10.6629 | 0.0011 | 90.62% |
| Germination rate | Licorice species | 8.6780 | 0.0032 | 88.48% |
|  | Experiment type | 6.2431 | 0.0125 | 83.98% |
|  | Drought method | 1.6621 | 0.1973 | 39.84% |
|  | Drought intensity | 2.6693 | 0.2632 | 25.07% |
|  | ExtraTreatment type | 4.0889 | 0.1295 | 51.09% |
| Germination potential | Licorice species | — | — | — |
|  | Experiment type | 4.0419 | 0.0444 | 75.26% |
|  | Drought method | 4.0419 | 0.0444 | 75.26% |
|  | Drought intensity | 10.9967 | 0.0041 | 81.81% |
|  | ExtraTreatment type | 4.0419 | 0.0444 | 75.26% |
| Height | Licorice species | 0.7916 | 0.3736 | 0.00% |
|  | Experiment type | 37.4695 | < 0.0001 | 97.33% |
|  | Drought method | 17.0139 | < 0.0001 | 94.12% |
|  | Drought intensity | 0.7065 | 0.7024 | 0.00% |
|  | ExtraTreatment type | 62.2782 | < 0.0001 | 95.18% |
| Root length | Licorice species | 2.3508 | 0.3087 | 14.92% |
|  | Experiment type | 74.9958 | < 0.0001 | 98.67% |
|  | Drought method | 24.9209 | < 0.0001 | 95.99% |
|  | Drought intensity | 0.1571 | 0.9244 | 0.00% |
|  | ExtraTreatment type | — | — | — |
| Root fresh weight | Licorice species | 0.0217 | 0.8828 | 0.00% |
|  | Experiment type | 14.5736 | 0.0001 | 93.14% |
|  | Drought method | 9.8359 | 0.0017 | 89.83% |
|  | Drought intensity | 2.7869 | 0.2482 | 28.24% |
|  | ExtraTreatment type | 13.6778 | 0.0011 | 85.38% |
| Shoot fresh weight | Licorice species | 5.2641 | 0.0218 | 81.00% |
|  | Experiment type | 14.1594 | 0.0002 | 92.94% |
|  | Drought method | 1.5870 | 0.2078 | 36.99% |
|  | Drought intensity | 5.3145 | 0.0701 | 62.37% |
|  | ExtraTreatment type | 24.0502 | < 0.0001 | 87.53% |
| Glycyrrhizic acid | Licorice species | 6.4370 | 0.0400 | 68.93% |
|  | Experiment type | 6.4746 | 0.0109 | 84.56% |
|  | Drought method | 1.0789 | 0.2989 | 7.31% |
|  | Drought intensity | 0.9768 | 0.6136 | 0.00% |
|  | ExtraTreatment type | 6.4370 | 0.0400 | 68.93% |
| Liquiritin | Licorice species | 3.3385 | 0.0677 | 70.05% |
|  | Experiment type | 3.3385 | 0.0677 | 70.05% |
|  | Drought method | 1.6548 | 0.1983 | 39.57% |
|  | Drought intensity | 1.4142 | 0.4931 | 0.00% |
|  | ExtraTreatment type | 4.1418 | 0.1261 | 51.71% |
| Total flavonoids | Licorice species | 23.7135 | < 0.0001 | 91.57% |
|  | Experiment type | 13.0852 | 0.0003 | 92.36% |
|  | Drought method | 25.1297 | < 0.0001 | 96.02% |
|  | Drought intensity | 0.6913 | 0.7077 | 0.00% |
|  | ExtraTreatment type | 37.0705 | < 0.0001 | 91.91% |
| Total phenols | Licorice species | 8.7016 | 0.0032 | 88.51% |
|  | Experiment type | 8.7016 | 0.0032 | 88.51% |
|  | Drought method | 8.7016 | 0.0032 | 88.51% |
|  | Drought intensity | 1.0517 | 0.5910 | 0.00% |
|  | ExtraTreatment type | 12.9453 | 0.0015 | 84.55% |
| SOD | Licorice species | 6.0641 | 0.0482 | 67.02% |
|  | Experiment type | 0.0263 | 0.8712 | 0.00% |
|  | Drought method | 6.9140 | 0.0086 | 85.54% |
|  | Drought intensity | 0.6413 | 0.7257 | 0.00% |
|  | ExtraTreatment type | 0.6136 | 0.7358 | 0.00% |
| POD | Licorice species | 9.1201 | 0.0105 | 78.07% |
|  | Experiment type | 6.5877 | 0.0103 | 84.82% |
|  | Drought method | 1.3787 | 0.2403 | 27.47% |
|  | Drought intensity | 1.5939 | 0.4507 | 0.00% |
|  | ExtraTreatment type | 16.0312 | 0.0003 | 87.52% |
| CAT | Licorice species | 2.1319 | 0.3444 | 6.19% |
|  | Experiment type | 9.6011 | 0.0019 | 89.58% |
|  | Drought method | — | — | — |
|  | Drought intensity | 1.3790 | 0.5018 | 0.00% |
|  | ExtraTreatment type | 11.6333 | 0.0030 | 82.81% |
| MDA | Licorice species | 3.9767 | 0.0461 | 74.85% |
|  | Experiment type | 3.9767 | 0.0461 | 74.85% |
|  | Drought method | 21.1925 | < 0.0001 | 95.28% |
|  | Drought intensity | 0.6697 | 0.7154 | 0.00% |
|  | ExtraTreatment type | 22.0319 | < 0.0001 | 90.92% |
| Proline | Licorice species | 7.9713 | 0.0186 | 74.91% |
|  | Experiment type | 0.2847 | 0.5936 | 0.00% |
|  | Drought method | 1.8742 | 0.3918 | 0.00% |
|  | Drought intensity | 2.8589 | 0.0909 | 65.02% |
|  | ExtraTreatment type | 0.0418 | 0.8379 | 0.00% |
| Soluble protein | Licorice species | 1.0601 | 0.5886 | 0.00% |
|  | Experiment type | 0.3346 | 0.5629 | 0.00% |
|  | Drought method | 0.0269 | 0.8698 | 0.00% |
|  | Drought intensity | 0.4698 | 0.7906 | 0.00% |
|  | ExtraTreatment type | 0.7538 | 0.6860 | 0.00% |
| Soluble sugar | Licorice species | 9.0148 | 0.0110 | 77.81% |
|  | Experiment type | 5.5075 | 0.0189 | 81.84% |
|  | Drought method | 1.9478 | 0.1628 | 48.66% |
|  | Drought intensity | 2.2366 | 0.3268 | 10.58% |
|  | ExtraTreatment type | 7.4786 | 0.0238 | 73.26% |

Note: I² was calculated as max[0, (QB - df) / QB × 100%]. A dash indicates that the corresponding subgroup heterogeneity result was not available in the provided text.

**Table S2. Output results of Rosenthal fail-safe number test for different groups.**

| **Variable** | **Fail-safe number** | **Standard value** | **N** | **Result** |
| --- | --- | --- | --- | --- |
| Overall | 125891.9 | 3490 | 696 | Robust |
| SOD | 872.3 | 225 | 43 | Robust |
| POD | 872.3 | 225 | 43 | Robust |
| CAT | 711.2 | 165 | 31 | Robust |
| MDA | 736.5 | 180 | 34 | Robust |
| Pn | 495.8 | 140 | 26 | Robust |
| Gs | 472.4 | 125 | 23 | Robust |
| Ci | 736.5 | 180 | 26 | Robust |
| Tr | 736.5 | 180 | 26 | Robust |
| Chl a | 412.8 | 100 | 18 | Robust |
| Chl b | 412.8 | 100 | 18 | Robust |
| SPAD | 481.3 | 130 | 24 | Robust |
| Shoot fresh | 846.7 | 200 | 38 | Robust |
| Root fresh | 833.3 | 195 | 37 | Robust |
| Root length | 701.9 | 155 | 29 | Robust |
| Height | 846.7 | 200 | 38 | Robust |
| Germination energy | 355.3 | 85 | 15 | Robust |
| Germination percentage | 696.1 | 175 | 33 | Robust |
| Liquiritin | 653.2 | 155 | 20 | Robust |
| Total flavonoids | 736.5 | 180 | 34 | Robust |
| Total Phenols | 859.0 | 210 | 40 | Robust |
| Glycyrrhizic acid | 753.2 | 190 | 36 | Robust |
| Pro | 458.9 | 115 | 21 | Robust |
| Soluble sugars | 423.6 | 105 | 19 | Robust |
| Soluble protein | 438.4 | 125 | 23 | Robust |

**Table S3.** Categorical variables and the subgroups within each variable

| Categorical variable | Subgroup | | |
| --- | --- | --- | --- |
| Licorice species | *Glycyrrhiza glabra* | *Glycyrrhiza uralensis* | *Glycyrrhiza inflata* |
| Experiment type | Indoor | Field |  |
| Drought method | NO | Ca^2+^ | Betaine |
| Drought intensity | Mild | Moderate | Severe |
| Licorice species | PEG-6000 | Water Control |  |
